# Validation of an implant stability measurement device using the percussion response: a clinical research study

**DOI:** 10.1186/s12903-022-02320-0

**Published:** 2022-07-14

**Authors:** Yurie Okuhama, Koudai Nagata, Hyunjin Kim, Hayato Tsuruoka, Mihoko Atsumi, Hiromasa Kawana

**Affiliations:** 1 Department of Oral and Maxillofacial Implantology, Kanagawa Dental University, 82 Inaoka-cho, Yokosuka, Kanagawa 238-8580 Japan; 2 Department of Fixed Prosthodontics, Kanagawa Dental University, 82 Inaoka-cho, Yokosuka, Kanagawa Japan

**Keywords:** Dental implant, Implant stability quotient (ISQ), Implant stability test (IST), AnyCheck®, Osstell®

## Abstract

**Background:**

Several devices have been developed to measure implant-bone stability as an indicator of successful implant treatment; these include Osstell®, which measures the implant stability quotient (ISQ), and the more recent AnyCheck®, which relies on percussion for the implant stability test (IST). These devices make it possible to measure implant stability. However, no studies have compared the performance of AnyCheck® and Osstell® (i.e., IST and ISQ values) in clinical practice. Therefore, this study aimed to determine the correlation between primary and secondary implant stability using the Osstell® and AnyCheck® devices.

**Methods:**

Ten patients (7 women; age [mean ± standard deviation]: 49.1 ± 13.3 years) with partially edentulous jaws who received a total of 15 implants were included. IST (AnyCheck®) and ISQ (Osstell®) values were measured immediately after implantation and at 1, 2, 3, 4, and 6 weeks post-implantation. Each measurement was performed three times, and the average value was used as the result. The correlation between measurements obtained using the two devices was determined using Spearman's rank correlation coefficient.

**Results:**

The IST values ranged from 79.1 ± 2.87 to 82.4 ± 2.65. The ISQ values ranged from 76.0 ± 2.8 to 80.2 ± 2.35. Spearman's rank correlation coefficient was r = 0.64 immediately after implantation, r = 0.29 at 1 week, r = 0.68 at 2 weeks, r = 0.53 at 3 weeks, r = 0.68 at 4 weeks, and r = 0.56 at 6 weeks. A positive correlation was found in all cases, except at week 1 when the correlation was weak; the IST and ISQ values decreased the most during the first postoperative week and increased during the second week. The IST values were also slightly higher at all measurement points.

**Conclusion:**

The ability to assess implant stability without removing the abutment during healing is essential for determining the timing of loading without the risk of bone resorption. The results of this study suggest that AnyCheck® is useful for determining primary and secondary implant stability.

## Background

In recent years, the use of dental implants has become widespread in the field of dentistry, and various technological advancements have been proposed to improve treatment outcomes [1–3]. For instance, several devices have been developed to measure implant stability as an indicator of the success of implant treatment. The Osstell® device [4] allows the measurement of the implant stability quotient (ISQ) using the resonance frequency analysis (RFA) method, whereas the Periotest® device [5] uses the percussion method. More recently, the AnyCheck® device, which also relies on the percussion method, has been developed [6]. Importantly, the insertion torque (IT) of the implant into the bone influences the success of implant treatment; therefore, the ability of these devices to quantify and evaluate implant stability has contributed greatly to the success of implant treatments [7, 8], benefitting both dentist and patients. There are two types of implant surgery: those that allow submerged implant healing and those with non-submerged implant healing. Submerged implant healing is often considered when the primary stability is poor or when bone grafting has been performed [9]. In non-submerged implant healing, removal of healing abutments prior to superstructure placement has been reported to be a cause of accelerated bone resorption [10]. Therefore, the concept of “one abutment–one time,” in which the abutment is placed immediately after implantation to control bone resorption, is popular [11]. Despite its long history of use, the Osstell® device requires removal of the healing abutment and the attachment of smart pegs. Of note, AnyCheck® does not require the healing abutment to be attached or removed; therefore, it can measure implant stability without promoting bone resorption. Although there have been various reports on implant stability, thus far, no study has compared the ISQ and implant stability test (IST) values in clinical practice [12]. To address this gap in knowledge, the present study aimed to investigate the correlation between implant stability for the Osstell® and AnyCheck® devices.

## Materials and methods

### Patients

Ten patients (7 women, 3 men) with partially edentulous jaws who underwent implant treatment at our university hospital (n = 15 implants) were included in this study. The mean age (± standard deviation) was 49.1 ± 13.3 years. Patients were selected based on absence of systemic diseases, smoking status (non-smokers), and non-requirement of bone grafting. The IT was set at 35 Ncm using micromotor and torque wrench for all patients. Healing abutments of the following diameters were attached to the implants: 2 mm in one implant, 4 mm in nine, and 6 mm in five implants. This study was approved by the institutional ethics committee of our hospital (approval #739), and written informed consent was obtained from all patients.

### Surgical procedure

All patients were instructed to take an oral dose (1 g) of amoxicillin hydrate (Sawacillin Capsules®; LTL Pharma, Tokyo, Japan) 1 h before surgery. After administration of the anesthetic (Lidocaine/Adrenaline bitartrate®; Showa Yakuhin Kako Co., Ltd., Tokyo, Japan), the alveolar mucosa, including the periosteum, was incised at the top of the ridge and separated. After drilling, implants were placed according to the implant system protocol; the torque and depth of placement were adjusted with a torque ratchet. All implant placements were performed via freehand insertion; additionally, all surgeries were performed in a non-submerged fashion. The implant system used was Straumann® SLActive φ 4.1 × 10 (bone level tapered implant; Basel, Switzerland). All surgeries were performed by the same doctor, a teaching Associate in the Department of Implantology at our university hospital.

### Measurement of the IST and ISQ values

**Fig. 1:**
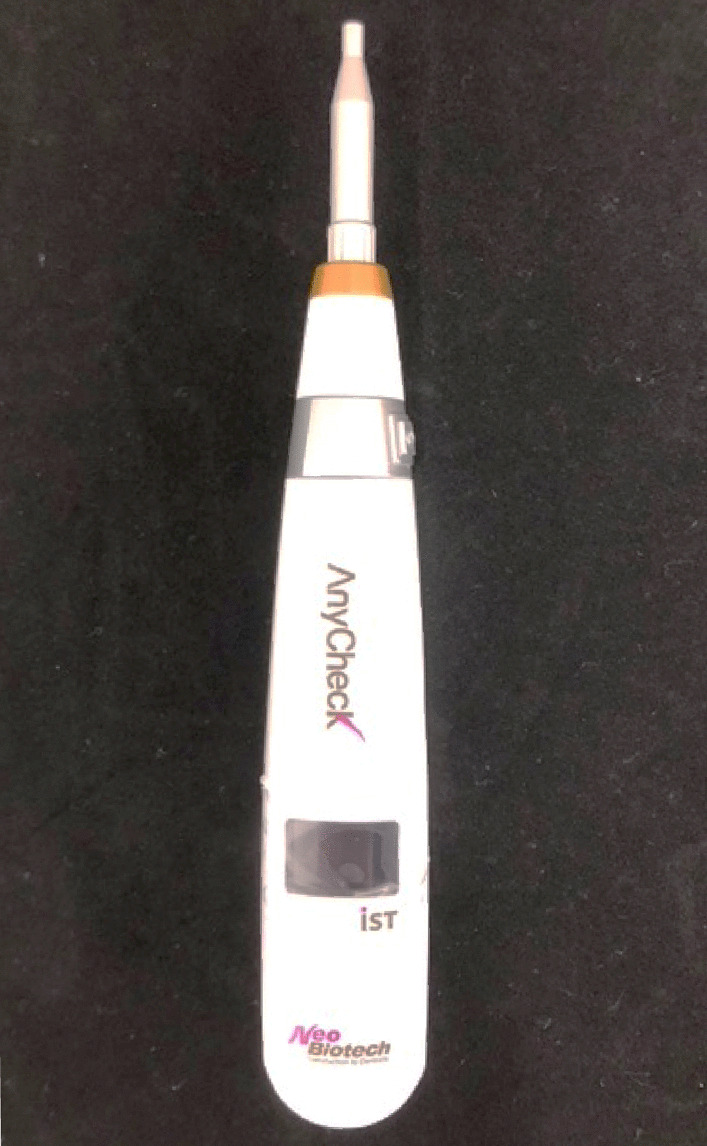
The AnyCheck® implant stability test (IST) device

The IST values were measured using the AnyCheck® device (Neobiotech Co., Ltd., Seoul, South Korea) (Fig. 1). The bone-to-implant stability index was set based on the ISQ values (0–59, not recommended for loading; 60–99, good stability, recommended for loading); the IST and ISQ have similar reference values. Osstell® was used instead of Periotest® in this study. Briefly, to determine the IST value, the healing abutment was struck six times over 3 s, and the contact time with the healing abutment was measured to calculate the stability. Notably, in accordance with the manufacturer’s recommendations, the patient was placed in an upright position during measurement, and the contact angle was set at 0°–30°. Since AnyCheck® uses a standard healing abutment height of 4 mm, values for healing abutment heights other than 4 mm were corrected as recommended by the manufacturer (Table 1).

**Table 1:** Corrected IST values, measured using the AnyCheck® device, based on the healing abutment height

Healing abutment height (mm)	IST value
7	+ 6
6	+ 4
5	+ 2
4	± 0
3	− 2
2	− 4
1	− 6

IST, implant stability test

**Fig. 2:**
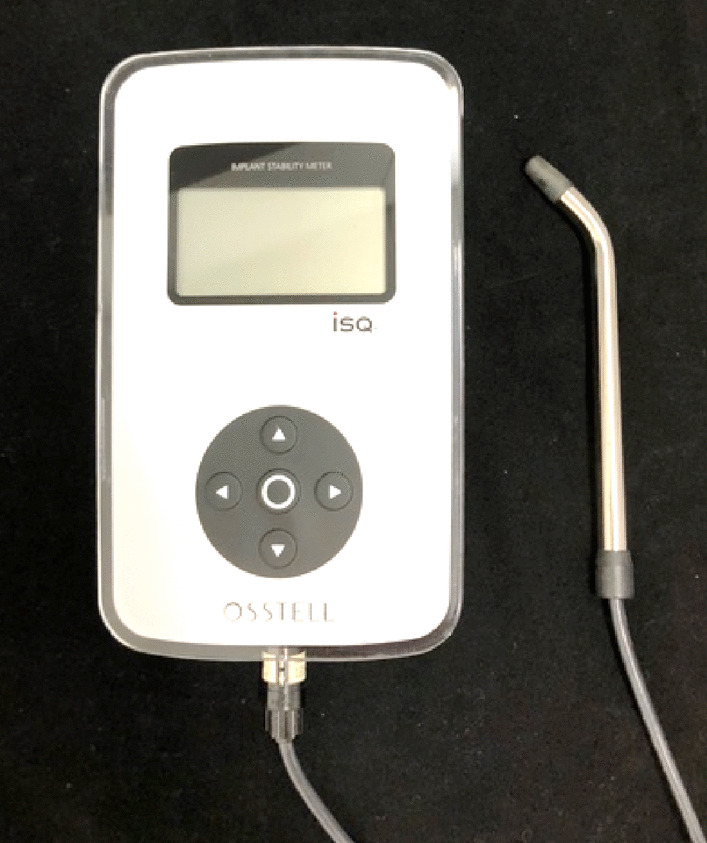
The Osstell® implant stability quotient (ISQ) device

The ISQ values were determined using the Osstell® ISQ device (Integration Diagnostics Ltd., Goteborgsvagen, Sweden) (Fig. 2). In principle, magnetic pulses based on the RFA method stimulate and resonate the smart peg (Integration Diagnostics Ltd.) attached to the implant body in the patient’s mouth, making it possible to quantify stability. At the time of measurement, the intraoral healing abutment was removed, and the smart peg was attached to the implant body via hand tightening.

Both the IST and ISQ values were measured immediately after implantation and at 1-, 2-, 3-, 4-, and 6-weeks post-implantation. Each measurement was taken three times, and the mean was used as the definitive result. The ISQ was measured following assessment of the IST. For all implants, impressions were obtained at 4 weeks after placement, and provisional restorations were placed at 6 weeks. All measurements were taken by the same dental surgeon.

### Statistical analyses

Correlations between the IST and ISQ values were assessed using BellCurve for Excel (Social Survey Research Information, Inc., Tokyo). Spearman's rank correlation coefficients were used to determine correlations.

Sample size was calculated by one-way analysis of variance using G-Power (version 3.1.9.2). The sample size required to obtain 80% of the effect size of 0.4 at α = 0.05 was calculated.

## Results

**Fig. 3:**
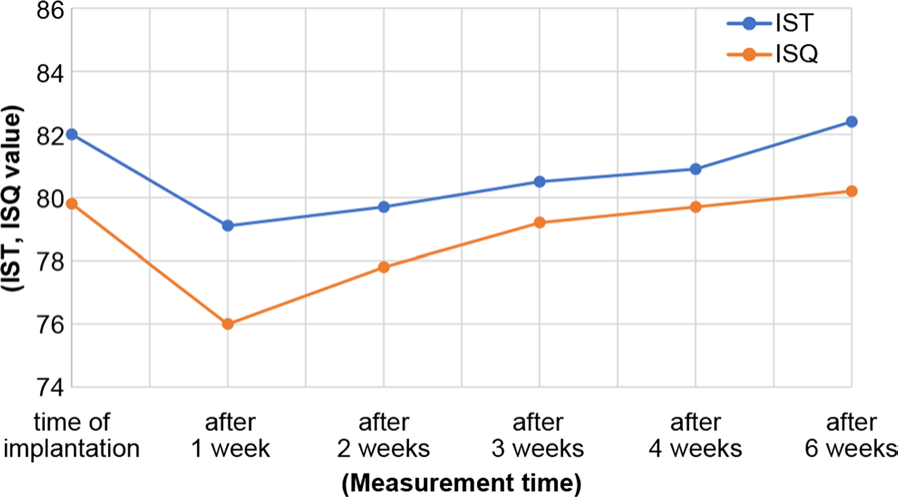
Comparison of the mean implant stability test (IST) and implant stability quotient (ISQ) values at different times post-implantation

**Fig. 4:**
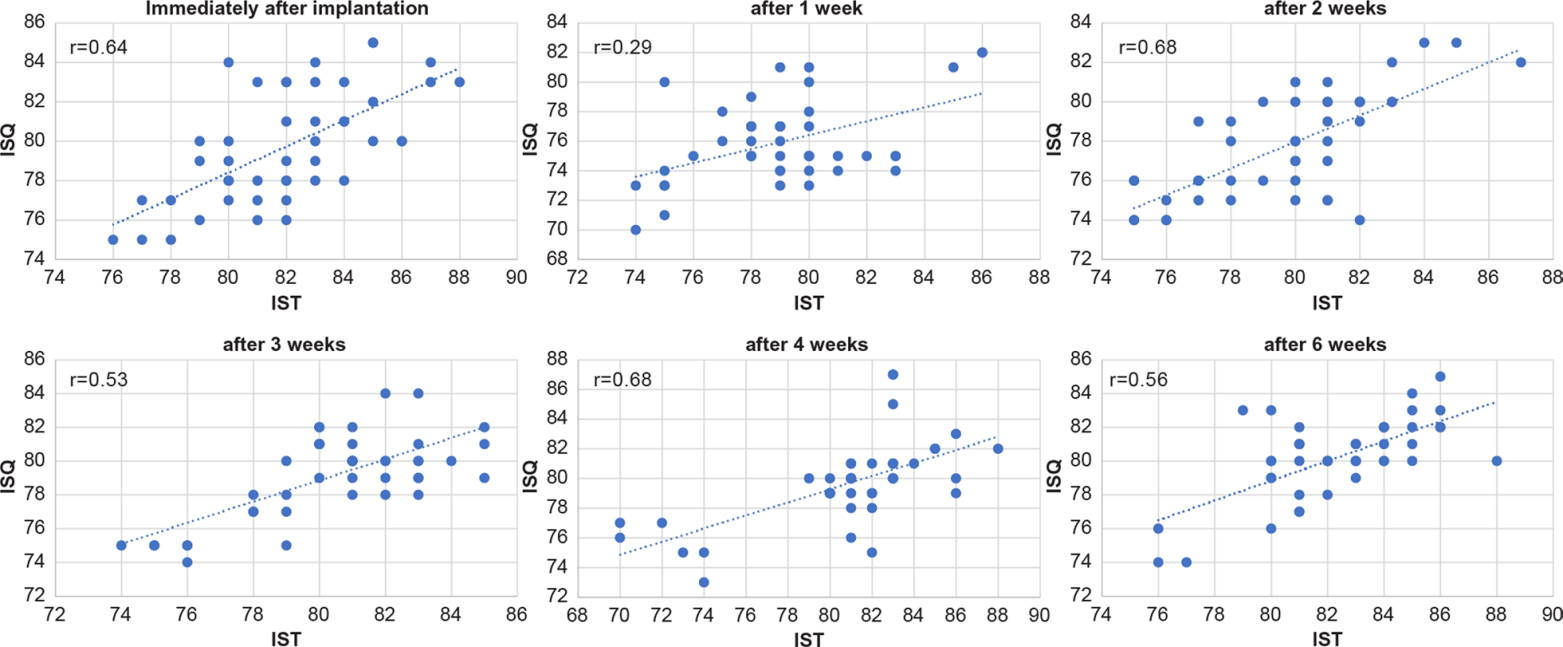
Correlation between the AnyCheck® and Osstell® at each measurement point

The IST values immediately, 1 week, 2 weeks, 3 weeks, 4 weeks, and 6 weeks after implantation were 81.0 ± 2.82, 79.1 ± 2.87, 79.7 ± 2.83, 80.5 ± 2.71, 80.9 ± 4.0, and 82.4 ± 2.65, respectively. The ISQ values immediately, 1 week, 2 weeks, 3 weeks, 4 weeks, and 6 weeks after implantation were 79.8 ± 2.89, 76.0 ± 2.8, 77.8 ± 2.63, 79.2 ± 2.44, 79.7 ± 2.77, and 80.2 ± 2.35, respectively (Fig. 3). Of note, both the IST and ISQ values decreased the most in the first week after surgery and increased in the second week; additionally, the IST value was slightly higher at all measurement points. The Spearman’s rank correlation coefficients for each measurement period were as follows: r = 0.64 immediately after implantation; r = 0.29 at 1 week; r = 0.68 at 2 weeks; r = 0.53 at 3 weeks, r = 0.68 at 4 weeks, and r = 0.56 at 6 weeks. A positive correlation was found in all cases, except at 1 week when the correlation was weak (Fig. 4).

## Discussion

This study compared the changes in implant stability using the Osstell® and AnyCheck® devices. Our analysis indicated that the measurements exhibited a positive correlation of > 0.5, except after 2 weeks. This suggested that AnyCheck® had the same performance as Osstell®.

When the IT is high, bone resorption is promoted. Optimization of the IT is considered the key to successful implant treatment [13–15]. In this study, all the implants had an IT of 35 Ncm. However, even in cases of low IT, the use of AnyCheck® allows safe assessment of implant stability. The IST and ISQ values in this study were high. Zwaan et al. [16] placed 163 implants in the maxilla and compared the IT at 50 Ncm, 40–45 Ncm, 30–35 Ncm, and ≤ 30 Ncm and found that the ISQ values were 76.2 ± 5.3, 72.3 ± 5.3, 70.0 ± 6.7, and 68.1 ± 6.2, respectively. The ISQ values were also reported to be higher for tapered implants than for straight implants. Van Eekeren et al. [17] compared bone-level with tissue-level implants and revealed that the ISQ values (at the time of placement and 2, 3, and 12 weeks postoperatively) were 77.8, 75.6, 76.3, and 79.1, and 74.0, 71.8, 72.6, and 76.8, respectively. Importantly, the above results suggest that ISQ values tend to vary according to bone quality, implantation site, and implant shape, in line with the findings reported elsewhere [18]. As reported above, the authors of this study think that the high value was due to the use of bone-level and tapered implants. Oates et al. [19] reported that the stability of SLActive® implants changed from a decrease to an increase at 2 weeks after placement, in line with our results. In the present study, the weakest correlation was observed after 2 weeks. This may be explained by individual differences in the decline of primary stability, resulting in large differences in IST and ISQ.

Park et al. [6] placed an implant into an artificial bone block to verify the accuracy of AnyCheck®; interestingly, the stability decreased as the height of the healing abutment increased and as the contact angle decreased from 30° to 0° (perpendicular to the long axis of the implant and parallel to the ground). Subsequently, Lee et al. [20] placed implants at 10 N, 15 N, and 35 N into artificial bone blocks together with five different diameters of healing abutments of the same height, measured the IST values using AnyCheck®, and compared them with the ISQ values determined using Osstell®. Importantly, they reported that the diameter of the healing abutment did not affect the ISQ and IST values, which exhibited a strong correlation. Consistent with these results, Lee et al. [21] also found that the results for the AnyCheck® and Osstell® devices were correlated in the context of both internal-connection and external-connection implants (within pig bone). Of note, they also reported that the IST values were higher for both implants and that there was no significant difference between the IST and ISQ values. However, neither the IST nor the ISQ values are known to be accurate; they should only be considered as one among several indicators.

In clinical practice, Al-Jamal et al. [22] demonstrated that there was a significant correlation between primary stability and IT using the AnyCheck® device in the context of 40 implants. However, they did not compare their findings with measurements obtained using the Osstell® device. The present study is the first in which the IST and ISQ values were measured and compared weekly in clinical practice, from immediately after implantation to 4 weeks later. While the Osstell® is a device with a long history of use and has been explored in many studies to date, its use requires removal of the healing abutment and attachment of the smart peg. The recently released Osstell Beacon® is cordless. However, as before, it still requires a smart peg, and the healing abutment must be attached and removed. Esposito et al. [23] reported that the removal of the healing abutment (three times after implantation until the time of superstructure attachment) led to 0.16 mm of bone resorption per year (*versus* non-removal of the healing abutment). Similar results were obtained by Bressan et al. [24]—0.43 mm of bone resorption over 3 years in healing abutment removal *versus* non-removal contexts—as well as by Koutouzis et al. [25]—0.13 mm versus 0.28 mm bone resorption in 6 months after implantation in the without versus with healing abutment removal context). Importantly, AnyCheck®, which allows the measurement of stability without the need to attach or to detach the healing abutment, reduces bone resorption and can be applied to low-torque cases. In the present study, a positive correlation of > 0.5 was observed at all measurement points, except after 2 weeks. Considering the risk of bone resorption and other factors, the AnyCheck® is expected to perform as well or better than the Osstell®. Since there are no reports comparing the two devices in clinical practice, further validation of this matter is necessary. Furthermore, this study has some limitations. The sample size for this study was small. This was due to the limited number of patients in whom implants of the same system, diameter, and length were placed. In addition, in vitro studies cannot assess changes in implant stability over time. Therefore, studies using models could not be conducted previously. In the future, it is necessary to distinguish between bone quality and implant diameter to obtain more detailed data.

## Conclusion

The ability to assess implant stability without removing the abutment during healing is essential for determining the time at which load can be applied without the risk of bone resorption. Altogether, our results suggest the similar performance of Osstell® and AnyCheck®, and, consequently, the usefulness of the latter for the determination of implant stability.

## Data Availability

The datasets generated and/or analyzed during the current study are not publicly available due to privacy and ethical concerns but are available from the corresponding author on reasonable request.

## Abbreviations

ISQ: Implant stability quotient
RFA: Resonance frequency analysis
IT: Insertion torque
IST: Implant stability test
